# Inflammatory and vascular placental lesions are associated with neonatal amplitude integrated EEG recording in early premature neonates

**DOI:** 10.1371/journal.pone.0179481

**Published:** 2017-06-23

**Authors:** Dorit Paz-Levy, Letizia Schreiber, Offer Erez, Sharon Goshen, Justin Richardson, VIadimir Drunov, Orna Staretz Chacham, Eilon Shany

**Affiliations:** 1 Department of Obstetrics and Gynecology, Soroka University Medical Center, Faculty of Health Sciences, School of Medicine, Ben-Gurion University of the Negev Beer Sheva, Israel; 2 Pathology Institute, Wolfson Medical Center, Hulon, Israel; 3 Department of Epidemiology, Faculty of Health Sciences, School of Medicine, Ben-Gurion University of the Negev, Beer Sheva, Israel; 4 Department of Neonatology, Soroka University Medical Center, Faculty of Health Sciences, School of Medicine, Ben-Gurion University of the Negev, Beer Sheva, Israel; 5 Department of Pathology, Soroka University Medical Center, Faculty of Health Sciences, School of Medicine, Ben-Gurion University of the Negev, Beer Sheva, Israel; Hungarian Academy of Sciences, HUNGARY

## Abstract

**Introduction:**

Placental histologic examination can assist in revealing the mechanism leading to preterm birth. Accumulating evidence suggests an association between intrauterine pathological processes, morbidity and mortality of premature infants, and their long term outcome. Neonatal brain activity is increasingly monitored in neonatal intensive care units by amplitude integrated EEG (aEEG) and indices of background activity and sleep cycling patterns were correlated with long term outcome. We hypothesized an association between types of placental lesions and abnormal neonatal aEEG patterns.

**Objective:**

To determine the association between the placental lesions observed in extreme preterm deliveries, and their neonatal aEEG patterns and survival.

**Patients and methods:**

This prospective cohort study included extreme premature infants, who were born ≤ 28 weeks of gestation, their placentas were available for histologic examination, and had a continues aEEG, soon after birth)n = 34). Infants and maternal clinical data were collected. aEEG data was assessed for percentage of depressed daily activity in the first 3 days of life and for sleep cycling. Associations of placental histology with clinical findings and aEEG activity were explored using parametric and non-parametric statistics.

**Results:**

Twenty two out of the 34 newborns survived to discharge. Preterm prelabor rupture of membranes (PPROM) or chorioamnionitis were associated with placental lesions consistent with fetal amniotic fluid infection (AFI) or maternal under perfusion (MUP) (P < 0.05). Lesions consistent with fetal response to AFI were associated with absence of SWC pattern during the 1^st^ day of life. Fetal-vascular-thrombo-occlusive lesions of inflammatory type were negatively associated with depressed cerebral activity during the 1^st^ day of life, and with aEEG cycling during the 2nd day of life (P<0.05). Placental lesions associated with MUP were associated with depressed neonatal cerebral activity during the first 3 days of life (P = 0.007).

**Conclusions:**

Depressed neonatal aEEG patterns are associated with placental lesions consistent with maternal under perfusion, and amniotic fluid infection of fetal type, but not with fetal thrombo-oclusive vascular disease of inflammatory type. Our findings highlight the association between the intrauterine mechanisms leading to preterm parturition and subsequent depressed neonatal cerebral function early after birth, which eventually may put premature infants at risk for abnormal neurodevelopmental outcome.

## Introduction

Spontaneous preterm parturition has been the concern of obstetricians and neonatologist in the past decades. Efforts of primary and secondary preventions of this syndrome with progesterone[1–6], cerclage[7–13] and pessary[14–17], yielded a decrease in the total rate of the preterm birth. However, when stratified according to gestational age, the rate of premature birth prior to 28 weeks of gestation did not change substantially during this period, and it varies around 1% of all deliveries[18].

The vast majority of early preterm deliveries are due to spontaneous premature parturition[19],a syndrome that is the clinical end point of many underlying mechanisms including microbial-induced inflammatory response, decidual hemorrhage and vascular disease, decidual senescence, disruption of maternal-fetal tolerances well as other mechanisms like uterine over distension and maternal stress[20–22].

The lack of success to decrease the rate of preterm delivery prior to 28 weeks of gestation may result from insufficient understanding of the underlying mechanisms leading to these severe obstetrical complications, hampering the ability to develop effective treatment modalities. The placenta reflects the underlying pathologies leading to very early premature delivery, and the study of these lesions may assist in the effort to prevent such early premature birth and in the treatment of theses neonates. Several studies in the recent years demonstrated correlations between premature parturition and histologic placental features of vascular and inflammatory lesions [23–31]. Moreover, in some of the cases with intra-amniotic infection and inflammation, the fetus is involved and a fetal inflammatory response syndrome can be detected[32–35]. These fetuses have an increased risk of abnormal neurodevelopmental outcome and CP later in their life.

Neonates delivered prior to 28 weeks of gestation have a substantially higher rate of short (broncho pulmonary dysplasia, necrotizing enterocolitis, respiratory distress syndrome, retinopathy of prematurity, intraventricular hemorrhage) and long term complications of prematurity (cerebral palsy (CP) and abnormal neurocognitive development). The rate of these complications is inversely related to the gestational age at delivery.[36–40]

In light of the association between prematurity, especially the early one, and long term neurodevelopmental sequela, neonatal brain activity monitoring was introduced to the Neonatal Intensive Care Units (NICUs). Indeed, in the last two decades, amplitude-integrated EEG (aEEG), a technique with graphics-based visual display of electric cerebral activity is increasingly used in NICU in order to monitor neonates at high risk for brain injury, especially extremely premature ones[41–44]. Moreover, previous reports demonstrated an association between depressed aEEG recording and adverse long term neurodevelopmental outcome in premature neonates [45–47]. Thus, we hypothesized that the intrauterine pathological processes that lead to preterm parturition prior to 28 weeks of gestation will be reflected in the type of placental lesions as well as in the characteristics of abnormal brain activity during the early neonatal period.

The objective of this study was to determine the association between very early preterm birth, placental histopathologic lesions and aEEG patterns in premature infants born before 28 weeks of gestation.

## Methods

### Study population

This prospective cohort study was conducted in the NICU and the department of Obstetrics and Gynecology of Soroka University Medical Center (Beer-Sheva, Israel) between 2008 and 2010 with retrospectively collected maternal data.

Newborn included in the study:1) Were born ≤ 28 weeks of gestation(according to the last menstrual period and/or US scan in the first trimester, or according to embryo transfer for IVF pregnancies); 2) survived resuscitation after delivery and had an aEEG monitoring that was initiated prior to 6 hours of age; and 3) had a placental specimen available for histologic examination. Excluded from the study were newborns with major central nervous system anomalies or chromosomal abnormalities. The study protocol was approved by the Soroka Medical Center ethics committee and informed consent forms were signed by guardians of recruited infants. Maternal data were collected retrospectively from medical records of labor and delivery admissions and prospectively from newborns' files. Maternal information included demographic data (age, ethnicity, parity, medical conditions not related to pregnancy), risk factors for preterm birth (assisted reproductive treatment (ART), multiple gestation, amniotic fluid amount disorders, pre-eclampsia), information regarding the course of delivery (membrane rupture and its duration, preterm uterine contraction, chorioamnionitis, placental abruption, betamethasone treatment, antibiotic treatment, non-vertex presentation, cesarean delivery), and bacterial cultures data from maternal blood, urine, vagina, cervix, uterine cavity and from the placenta).

### Clinical definitions

Parity groups were defined as follows: nulliparous (1st delivery) multipara(deliveries ≥ 2). Amniotic fluid volume was estimated with a real time scanner equipped with a 3.5/5 MHz transducer of appropriate focal length. Oligohydramnios was defined as AFI < 5 [48] while polyhydramnios was defined as amniotic fluid index (AFI)>25 cm or a measurement of a maximal vertical pocket of at least 8 cm[49]; premature prelabor rupture of membranes (PPROM) was defined as any verified rupture of the chorioamniotic membranes before the onset of labor[50]. Chorioamnionitis was defined according to Gibbs criteria[51], preterm uterine contractions were defined according to maternal clinical presentation and confirming non stress test monitoring. Maternal temperature > 38°C that developed at least 24 hours after delivery, recorded by two different measurements at least four hours apart, or one measurement of maternal temperature of > 38.5°C, regardless of the time after delivery, was defined as post-partum fever. Wound infection was defined according to either clinical signs of infection or positive wound culture. Placental abruption was defined according to clinical placental examination after the third stage of labor. Betamethasone treatment was considered as given if at least one dose was administered before delivery. Antibiotic treatment was given to mothers who had PPROM before completed 34 weeks of gestation [52].

### Placental histology

Slides from placental paraffin blocks were retrieved from the pathology department. From each placenta, five tissue samples were used for histopathologic examination as follows: a slide from the free membranes (chorion and amnion with attached decidua capsularis), a slide from a section of the umbilical cord and three full thickness disc samples. In addition, any abnormal area was sampled. All samples stained with hematoxylin and eosin. Slides were analyzed by a single pathologist using a light microscope and classified into one of four groups according to Redline at al.[53]: Normal placenta, finding consistent with amniotic fluid infection (AFI), finding consistent with maternal under perfusion (MUP) or finding consistent with fetal vascular thrombo-occlusive disease (FVTOD).

### Neonatal data

Data were collected prospectively and included demographic data, delivery data (e.g. birthweight, Apgar score, cord blood pH), clinical hospitalization data (e.g. respiratory status, presence of sepsis and antibiotic treatment). Small for gestational age (SGA) was defined if newborn birthweight was below the 10th percentile, according to the revised Fenton growth curves[54]. Apgar score was dichotomized at<5 at 1 minute and <7 at 5 minutes, cord pH was dichotomized at cord blood pH<7.0. Respiratory distress syndrome (RDS), sepsis, convulsions, necrotizing enterocolitis (NEC), and intraventricular hemorrhage (IVH) were documented.

Brain ultrasound scans were carried out daily in the first three days of life, at one week of age, 30 and 34 weeks post conception. Short and long term outcome data were collected and included cerebral pathologies, presence of bronchopulmonary dysplasia and if the infant survived hospitalization, whether his neurological examination was within normal limits or not at discharge. Long term outcome at 2 years of age included a neurological examination [55–58] and Bayley's screening examination[59,60].

### aEEG recordings

As part of our local protocol, infants born at ≤28^th^ weeks of gestation are monitored with aEEG as soon as possible, and continued as clinically necessary. Informed consent for the incorporation of the information obtained from the aEEG in the study was sought from guardians of infants as soon as possible following the delivery. Enrolled infants were monitored for at least 72 hours after birth or until neonatal death.

**Fig 1:**
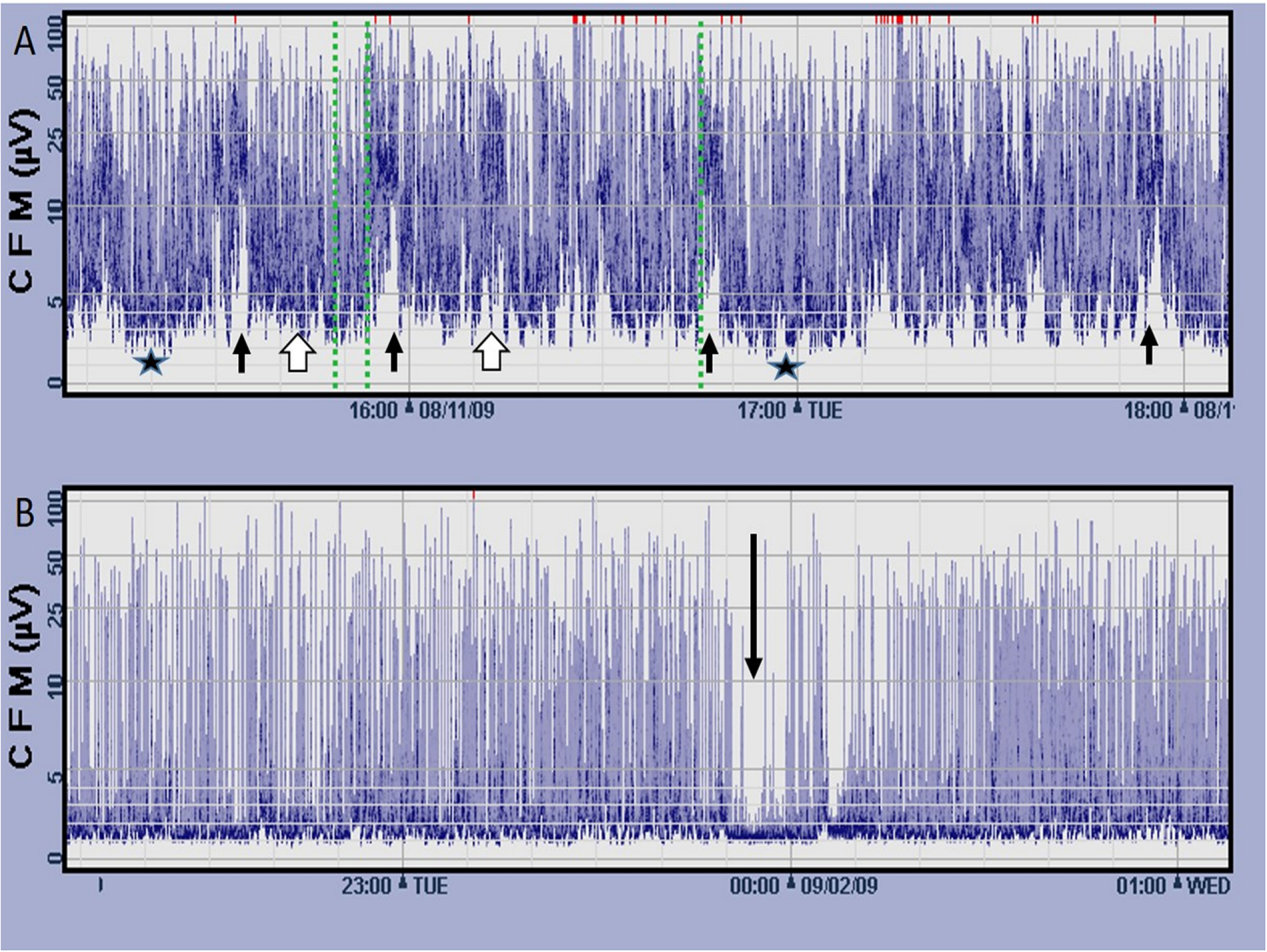
Patterns of aEEG recordings. A: Premature infants born at 27 weeks gestation at his first day of life. Normal tracing for age, note the cycling of the lower border of the aEEG tracing. Different patterns can be depicted in this tracing: Low discontinuous (black stars), high discontinuous (white arrows) and also short periods of continuous activity: black arrows. B: Premature infants bore 27 weeks gestation at his first day of life. Depressed tracing consisting of burst suppression pattern with a short period of isoelectric pattern (arrow).

aEEG was recorded with the CFM 6000 recorder (Olympic Medicals/Natus, Seattle, WA). In this method EEG signals are sampled from two parietal electrodes located at P3 and P4 areas according to the international 10/20 EEG system[61]. Frequencies under 2 and over 15 Hz are asymmetrically filtered after initial pre-amplification then the EEG signal is rectified, smoothened, whitened and compressed to a semi logarithmic scale. The final output, reflecting the maximum and minimum amplitudes of the original EEG, is displayed at speed of 6 cm per hour[62,63]. Pattern of each ten minutes segment of the aEEG recording was classified according to Olischaret. al. [41]: 1. Isoelectric, 2. Burst suppression, 3. Low discontinuous (baseline below 3 microvolts), 4. High discontinuous (baseline between 5 and 3 microvolts), 5.Continuous, and 6.Artifact. The percentage of daily pattern was computed and data was dichotomized to: Daily percentage of depressed aEEG (patterns 1, 2, and 3)) vs less depressed aEEG (patterns 4 and 5). Moreover, daily presence of cyclicity and seizure activity[64]was noted (Fig 1).

### Statistical analysis

Continuous data were presented as mean ± standard deviation (SD). Ordinary data were presented as median and inter-quartile range. Categorical data were presented as proportions. The normality of the aEEG activity was investigated by normal distribution attributes, skewness, kurtosis and inverse normal plot. Due to abnormal distribution, we have examined the associations of placental histology with maternal or neonatal clinical findings and aEEG activity by Mann-Whitney test for continuous variables. Chi Square or Fisher's exact test were used to assess categorical variables as appropriate.

In order to assess the first three days of life as a block, we divided our study group into three equal sub-groups (thirtiles) based on rate of depressed aEEG activity during the first days of life. Then we examined the associations between placental pathologies and depressed brain activity using the Mann Whitney test.

Differences were considered statistically significant when p<0.05. Data were analyzed using the Statistical Package for Social Sciences (IBM SPSS version 21).

## Results

During the study period 132 newborns were born at or prior to28 weeks of gestation. aEEG recording was initiated prior to 6 hours of age in 54 infants and 34 placental samples were available for histologic examination and consisted the study cohort.

### Clinical characteristics

The maternal and neonatal clinical characteristics are summarized in Table 1. The mean maternal age was 29 years, a third were nulliparous and 90% of them conceived following ART. The mean gestational age of the cohort was 26 weeks, 62% of the newborns survived to discharge.

**Table 1:** Maternal and neonatal clinical characteristics.

**Maternal and obstetrical Characteristics (n = 31)**	
Gravidity	2 (3.5)
Parity	1 (3)
Maternal age, (years)	29.09 ±6.28
Non-Jews (Bedouins)	18 (52.9%)
Preeclampsia Toxemia	2 (6%)
Antibiotics in labor	27 (79%)
Vaginal positive streptococcus group B	7 (20.6%)
Antenatal steroids:	24 (70.6%)
Magnesium sulfate	1 (2.9%)
Cesarean section	18 (58.06%)
**Neonatal Characteristics (N = 34)**	
Gestational age, weeks (mean ±SD)	26.07±1.28
Female	38.2%
Birthweight, gram (mean ±SD)	835.00±197
Head Circumference,cm (mean ±SD)	25.30±1.8
Intra-uterine growth, SGA	3 (8.82%)
Apgar 1' < 5	20 (58.82%)
Apgar 5' < 7	14 (41.17%)
Small for Gestational Age	3 (9%)
Survival at discharge	21 (62%)
Survival at 2 years of corrected age	20 (59%)
Umbilical Cord PH < 7.00	1 (3%)

Variables are presented as median (interquartile range), mean±standard deviation or N (%) as appropriate

### Placental lesions

**Fig 2:**
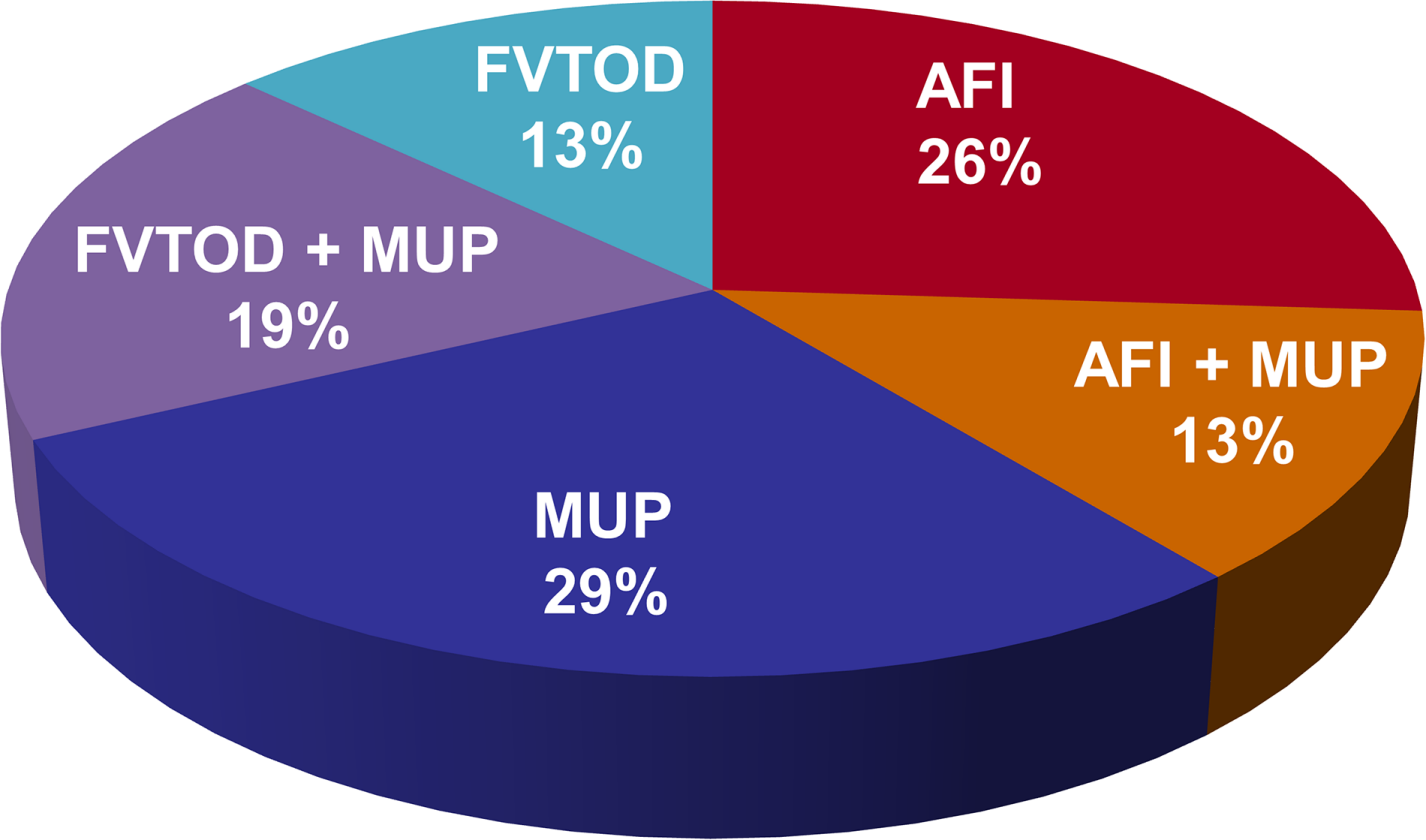
The distribution of placental lesions among cases. AFI- Amniotic Fluid Infection; MUP-Maternal Under Perfusion; FVTOD-Fetal Vascular Thrombo-Occlusive Disease.

**Fig 3:**
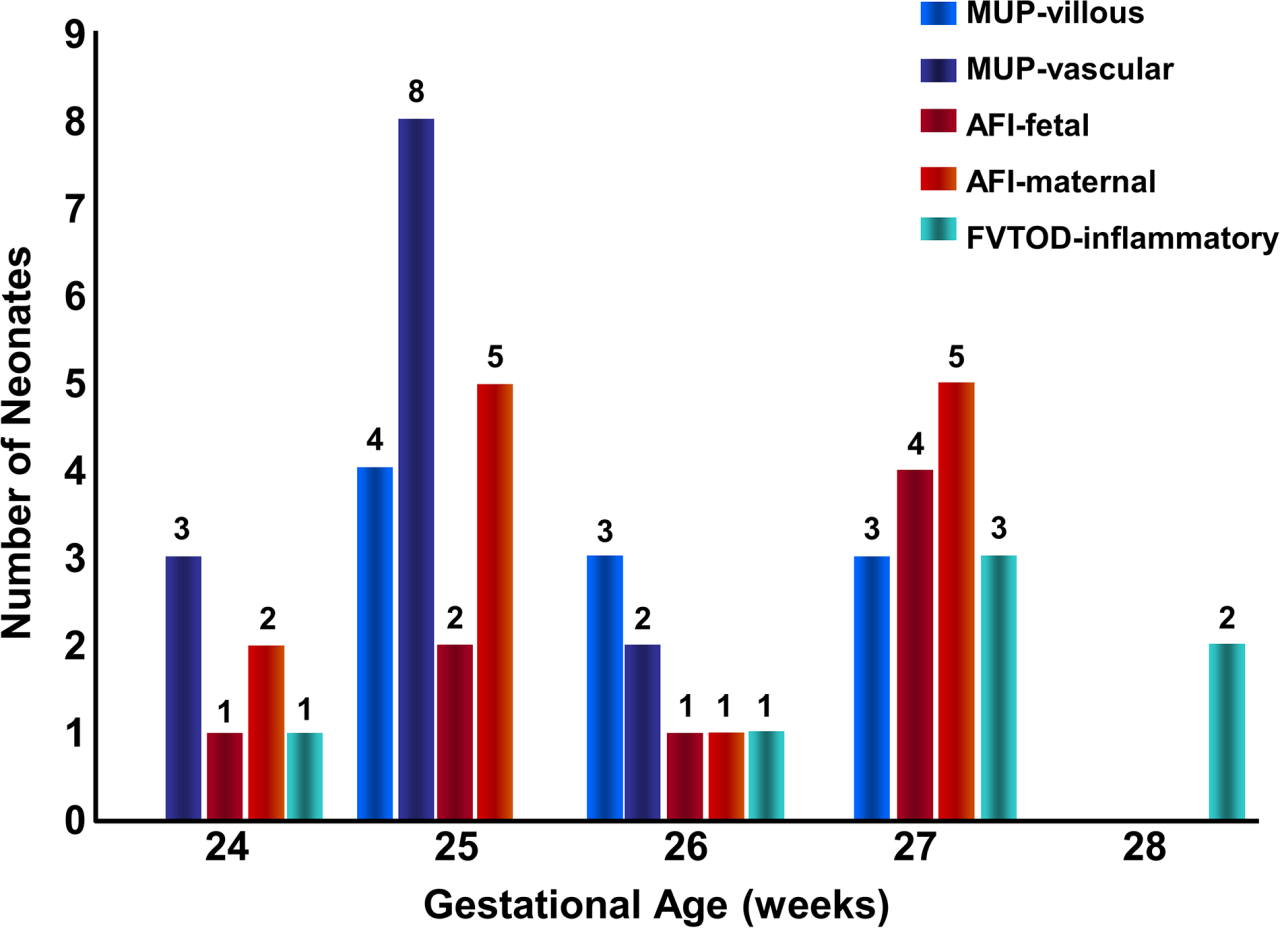
Association between placental histology and depressed aEEG—According to the neonatal percentage of daily depressed aEEG during the first three days of life. Significant association is demonstrated between MUP of a vascular type and neonates with the most depressed aEEG recording during the first 3 days of life. AFI- Amniotic Fluid Infection; MUP-Maternal Under Perfusion; FVTOD-Fetal Vascular Thrombo-Occlusive Disease.

Placental lesions were identified in all the 34 samples (Fig 2). In one third of the cases more than one type of placental of lesion was detected. Distribution of lesions according to gestational age is presented in Fig 3.

### The association of maternal and neonatal clinical presentation with placental lesions

Women with PPROM or with chorioamnionitis had a significantly higher prevalence of placental lesions associated with AFI (p = 0.001), and significantly lower rates of MUP(p = 0.038). None of the neonatal clinical variables including Apgar score, cord pH, rates of RDS, IVH, NEC, BPD and mortality were associated with a specific placental lesion.(Table 2).

**Table 2:** Maternal and neonatal clinical characteristics and placental oathology.

	Amniotic Fluid Infection (n = 12; 38.7%)		Maternal Under-Perfusion (n = 19; 61.3%)		Fetal Vascular Thrombo- Occlusive Disease (n = 10; 32.25%)	
**Maternal Clinical Presentation**						
	no. (%)	P value	no. (%)	P value	no. (%)	P value
Pre Eclampsia Toxemia (n = 2)	1 (50%)	NS	1 (50%)	NS	1 (50%)	NS
Oligohydramnios (n = 2)	1 (50%)	NS	2 (100%)	NS	0	NS
Polyhydramnios (n = 4)	2 (50%)	NS	3 (75%)	NS	2 (50%)	NS
Placental Abruption(n = 5)	2 (40%)	NS	3 (60%)	NS	1 (20%)	NS
PPROM (n = 8)	7 (88%)	0.001	2 (25%)	0.038	1 (12.5%)	NS
Preterm Uterine Contractions (n = 19)	6 (31%)	NS	12 (63%)	NS	6 (31%)	NS
Chorioamnionitis (n = 3)	3 (100%)	0.049	0	0.049	0	NS
**Neonatal Clinical Characteristic**						
	no. (%)	P value	no. (%)	P value	no. (%)	P value
SGA (n = 3)	0	NS	2 (66%)	NS	1 (33%)	NS
Ph of cord blood at delivery < 7.0 (n = 1)	1(100%)	NS	1(100%)	NS	0(0%)	NS
Apgar score 5 min < 7 (n = 14)	5 (35%)	NS	11(78%)	NS	3 (21%)	NS
Celeston prior to delivery (n = 24)	10 (41.7%)	NS	15 (62.5%)	NS	7 (29.2%)	NS
Magnesium prior to delivery (n = 1)	0(0%)	NS	1(100%)	NS	1(100%)	NS

Data is presented as n(%);PPROM: premature prelabor rupture of membranes

#### Associations of placental histology with neonatal cerebral activity

On the first day of life while cycling was not detected in 50% of infants with placental lesions consistent with AFI of the fetal side, it was recorded in all infants who had placental lesions consistent with FVTOD and with villous type MUP (p = 0.037). On the second day of life cycling was not detected in46%of infants with placental lesion consistent with AFI of the maternal side, 62.5% of those with AFI of the fetal side, 62% of those with MUP of vascular type and 57% of those with MUP of villous type, in contrast to lack of cycling in 20% of infants who had placental lesions with FVTOD of inflammatory type(p = 0.024) (Table 3).

**Table 3:** Association between placental histology and absence of sleep cycling.

	Neonatal day of life		
Placental Histology	Day 1 n = 7	Day 2 n = 17	Day 3 n = 13
**AFI maternal (n = 13)**	5 (38%)	6 (46%)	5 (38%)
**AFI fetal (n = 8)**	4 (50%) *	5 (62.5%)	3 (37.5%)
**MUP vascular (n = 16)**	3 (19%)	10 (62%)	6 (37.5%)
**MUP villous (n = 7)**	0 (0%)	4 (57%)	3 (43%)
**FVTOD—Inflammatory (n = 10)**	0 (0%)	2 (20%) **	4 (40%)

**Fig 4:**
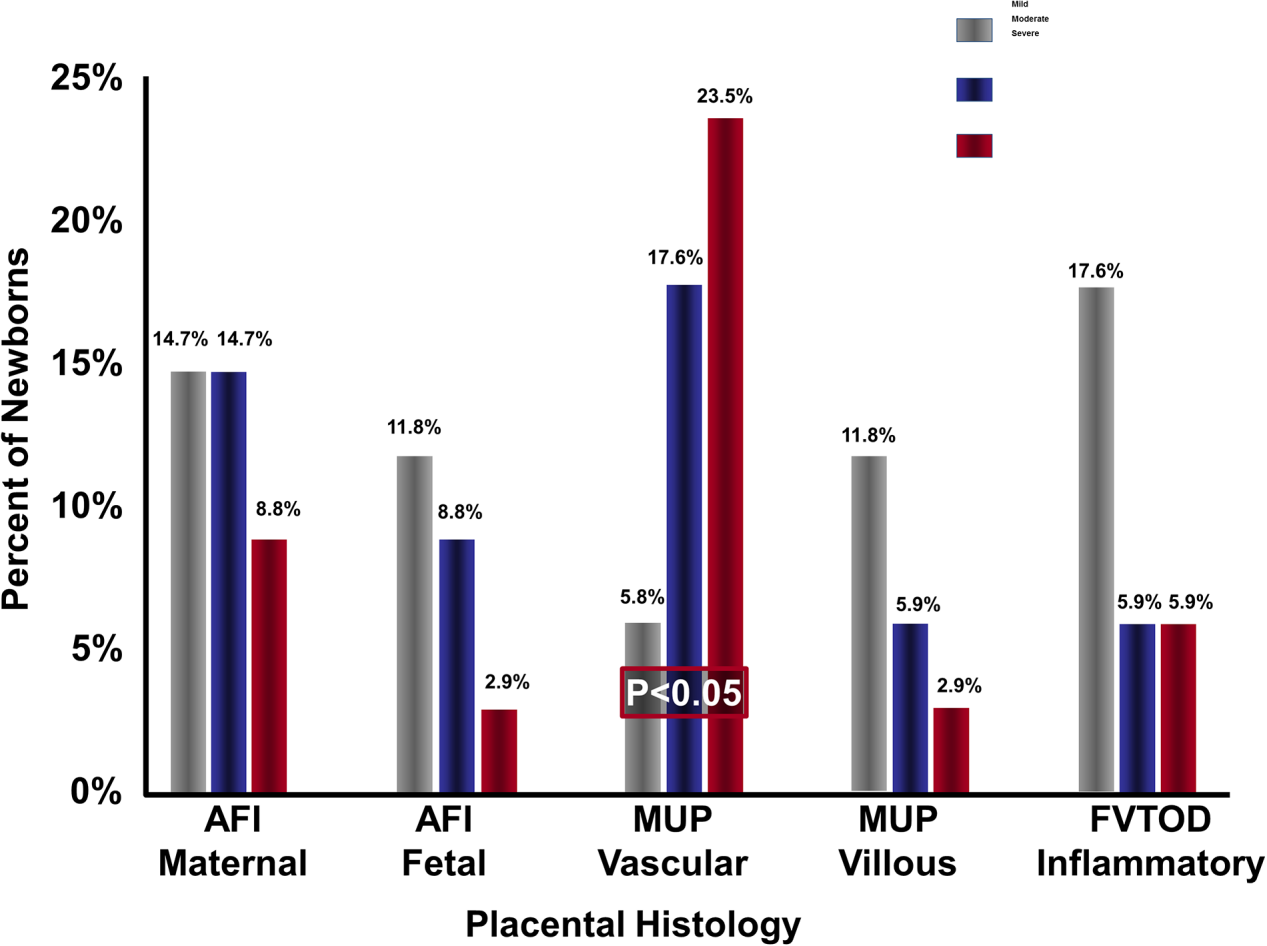
Distribution of placental lesions within thirtiles of cerebral activity. Infants with placental lesions consistent with vascular MUP were more likely to be in the highest thirtile of depressed cerebral activity. AFI: Amniotic Fluid Infection. MUP: Maternal Under Perfusion. FVTOD: Fetal Vascular Thrombo-Occlusive Disease.

**Fig 5:**
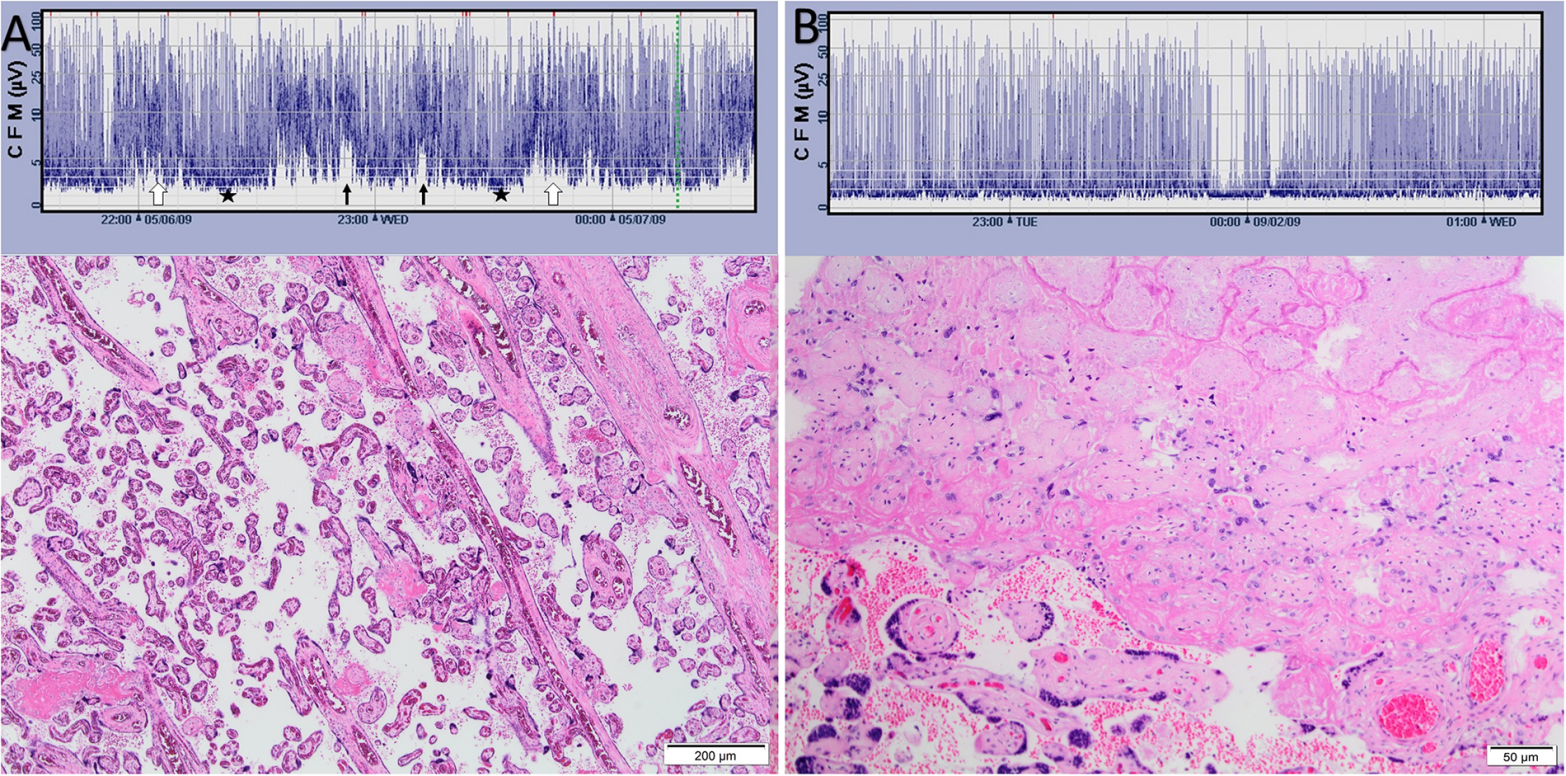
Patterns of aEEG recordings. A: Premature infants born 27 weeks' gestation at his first day of life. Normal tracing for age, note the cycling of the lower border of the aEEG tracing. Different patterns can be depicted in this tracing: Low discontinuous (black stars), high discontinuous (white arrows) and, also, short periods of continuous activity: black arrows. On histology, fetal vascular thrombo-occlusive type lesion was observed and he had a good outcome. B: Premature infants born 27 weeks' gestation at his first day of life. Depressed tracing consisting of burst suppression pattern with a short period of isoelectric pattern (arrow). In his placenta signs of maternal underperfusion, vascular type, were observed. As to his outcome, his Bayley's screening assessment classified him at risk and the Amiel-Tison neurological exam scored moderately abnormal.

AFI = Amniotic Fluid Infection; MUP = Maternal Under Perfusion; FVTOD = Fetal Vascular Thrombo-Occlusive Disease; data presented as rate(%);

* P Value = 0.037;

**p value = 0.024;

Infants with placental lesions consistent with MUP had a higher percentage of depressed cerebral activity in the first two days of life (day 1: p = 0.009, and day 2: p = 0.012) in comparison to those without these lesions. In addition, infants with placental lesions consistent with vascular MUP were more likely to be in the highest thirtile of depressed cerebral activity in regards to the overall percentage of depressed activity during the first three days of life, (p<0.05)(Fig 4). Neonates with placental lesions consistent with inflammatory FVTOD had a significantly lower percentage of time with depressed aEEG recording in the first day of life, as compared to those without these lesions (p = 0.016)(Table 4). Infants with mix placental lesions did not have a significant association with abnormal EEG patterns. (Fig 5)

**Table 4:** Daily percentages of depressed infant brain activity according to placental histology.

Placental lesion		Day of life					
		Day 1	P value	Day 2	P value	Day 3	P value
AFI maternal	Present	82±10	0.753	81±10	0.292	71±20	0.600
	Absent	78±20		75±20		70±20	
AFI fetal	Present	82±10	0.436	82±10	0.177	74±20	0.191
	Absent	74±20		69±20		61±20	
MUP vascular	Present	71±20	**0.009**	70±20	**0.012**	64±30	0.187
	Absent	89±10		89±10		79±10	
MUP villous	Present	81±10	0.379	80±10	0.771	73±20	0.427
	Absent	73±20		73±20		61±30	
FVTOD inflammatory	Present	84±10	**0.016**	81±20	0.061	72±20	0.867
	Absent	69±10		72±16		68±20	

AFI = Amniotic Fluid Infection; MUP = Maternal Under Perfusion; FVTOD = Fetal Vascular Thrombo-Occlusive Disease; data presented as mean ±standard deviation

## Discussion

### Principal findings of the study

Among early premature infants (≤28 weeks of gestation) platacental lesions consistent with intrauterine infection with fetal involvement were associated with absent aEEG cycling in the first day of life. In addition, placental lesions consistent with vascularMUPwere associated with depressed cerebral activity during the first two days of life and an overall lower cerebral activity during the first three days of life. In contrast, placental lesions consistent with FVTOD of inflammatory type wereless likely to be associated with abnormal cerebral activity.

### The association between adverse intrauterine events and neonatal morbidity of severe premature neonates

The current paradigm supports the concept that pathological processes leading to early premature delivery affect the fetus and the neonate[21,33–35,40]. Indeed, the involvement of the fetus in the pathological process leading to early premature birth is associated with the fetal inflammatory response syndrome (FIRS)[21,33–35,40], this condition is akin to the adult systemic inflammatory response syndrome observed during sepsis[21,33,34]. Recently FIRS was divided into two distinct phenotypes[65], the first results from intra-amniotic infection the affected also the fetus and is characterized by placental lesions consistent of amniotic fluid infection with fetal involvement (i.e. funisitis), and the second is characterized with maternal anti-fetal rejection which includes placental lesions associated with MUP of vascular type and chronic inflammatory lesions of the placenta. Both type of FIRS are associated with increased neonatal morbidity [32,66].

### The association between placental lesion and infant's neurodevelopment outcome

Prior reports suggested associations between specific placental lesions and short, as well as long, term neonatal neuro-developmental outcome [40,63,67–71]. Redline et. al.[70]reported that among extremely low birth weight infant, fetal vascular villus edema (a subtype of FVTOD), was associated with abnormal neurocognitive testing at school age, while maternal vascular lesions including increased syncytial knots (which is demonstrated in cases of MUP), were associated with subsequent development of CP, and fetal inflammatory response—as an expression of placental AFI on the fetal side, was associated with low mean neurocognitive score[70]. Van Viletet. al. found that infants with MUP had poorer mental development than very preterm infants with histological expression of AFI at 2 years of age[63]. Roescher et. al.[71]performed a systematic review of the association between placental histologic lesions, perinatal death, neonatal outcome and neurological development. They demonstrated an association between placental lesions of ascending intrauterine infection (AFI) with poor neurological outcomes. In addition, compared to gestational age—matched controls, infants born to mothers with placental findings consistent with maternal floor infarction,(a type of MUP), had a higher incidence of central nervous system injury in neonatal cranial ultrasound, and were more likely to have a suspicious or abnormal neurologic examination, lower developmental scores, and neurodevelopmental impairment[71]. Collectively these findings suggest that specific placental lesions are associated with subsequent short and long term neuro-developmental morbidity.

### Vascular placental lesions, severe prematurity, and depressed cerebral activity in early post-natal life—A variant of severe placental vascular disease?

In our cohort, unlike other reports [72,73] the most prevalent lesion was MUP (61%). The proportions of the specific lesions vary with the gestational age and classifications used [72,73]. Thus, Lee et. al.[72] reported a rate of 56% for acute chorioamnionitis (similar to Redline's AFI),7.4% for MUP, 4.4% for FVTOD, 1.5% for VUE 1.5% and 7.4% for chronic chorioamnionitis, Nijman et al. [73] reported 40.0% for placental infectious lesions and about 20% for MUP for this gestational age. The differences between the studies may result from different population, selection criteria and classifications.

Placental vascular MUP was the most common lesion in our study, and was associated with depressed aEEG recording during the first three days of life. This type of placental lesion is derived from implantation disorders and vascular remodeling, and is classically associated with pre-eclampsia and IUGR. Earlier reports found this lesion as more common among late preterm deliveries. However MUP is associated with subsequent development of CP in infants with extremely low birth weight[62].

Indeed, early onset preeclampsia, and IUGR are associated with increased risk for CP. Nevertheless, even in absence of these obstetrical complications placental vascular lesions that are associated with such early premature delivery may represent a higher degree of severity of the placental vascular disease that is usually observed later on during gestation when these lesions are more prevalent. Moreover, such severity of vascular disease can also affect the fetus and the neonate. Our novel finding that depressed aEEG recording during the first 3 days of life of extremely premature neonates are more prevalent in infants who had placentas with vascular MUP, support this view.

### Placental inflammatory lesions of the fetus and abnormal brain activity of the neonate

Placental lesions consistent with AFI of the fetal side of the placenta (i.e. funisitis) were associated in our population with absence of aEEG cycling on the first day of life. Previous reports found an association between the absence of neonatal aEEG cycling and abnormal neurodevelopmental outcome [45,46]. Impaired cerebral activity, as reflected by depressed activity or absence of cycling in aEEG recorded during the first 3 days of life, was reported by different researchers using different methodologies, to be predictive of white matter injury, short and long term adverse outcomes[45–47].

This small prospective cohort bring forth for the first time a possible link between specific placental lesion and abnormal infant brain function recording during the first three days of life. This suggests that the placenta may mirror the process leading to early prematurity and may assist to identify the newborns at risk for subsequent abnormal neurocognitive development and even CP. This information is important since it may have a role in early identification of neonates who will benefit from specific intervention to ameliorate the cerebral damage associated with early premature delivery.

### Strengths and limitations of the study

The major strength of our study is its prospective nature and our ability to demonstrate the association between clinical obstetrical conditions related to extreme prematurity, specific placental lesions and abnormal infant cerebral activity in the first days of life.

The small sample size and limited availability of placentas for histopathological evaluation restrained us from performing a multivariable analysis to strengthen the validity of our findings. Furthermore, though short and long term outcome of this cohort are available, we were not able to assess the associations between placental findings, cerebral activity and outcome.

### Conclusions

The association between abnormal neonatal cerebral activity and placental lesions in early premature neonates is novel; highlighting a possible association between intra uterine processes leading to extreme preterm parturition and depressed cerebral function early after birth which eventually put premature infants at risk of subsequent abnormal neuro-developmental outcome.

## References

[1] Conde-Agudelo A, Romero R (2016) Vaginal progesterone to prevent preterm birth in pregnant women with a sonographic short cervix: clinical and public healt implications. Am J Obstet Gynecol 214: 235–242. doi: 10.1016/j.ajog.2015.09.102 26450404 10.1016/j.ajog.2015.09.102 PMC5703061

[2] Hassan SS, Romero R, Vidyadhari D, Fusey S, Baxter JK, et al (2011) Vaginal progesterone reduces the rate of preterm birth in women with a sonographic short cervix: a multicenter, randomized, double-blind, placebo-controlled trial. Ultrasound Obstet Gynecol 38: 18–31. doi: 10.1002/uog.9017 21472815 10.1002/uog.9017 PMC3482512

[3] Romero R, Conde-Agudelo A, El-Refaie W, Rode L, Brizot ML, et al (2017) Vaginal progesterone decreases preterm birth and neonatal morbidity and mortality in women with a twin gestation and a short cervix: An updated meta-analysis of individual patient data. Ultrasound Obstet Gynecol. 10.1002/uog.17397 PMC5396280 28067007

[4] Romero R, Nicolaides KH, Conde-Agudelo A, O'Brien JM, Cetingoz E, et al (2016) Vaginal progesterone decreases preterm birth ≤ weeks of gestation in women with a singleton pregnancy and a short cervix: an updated meta-analysis including data from the OPPTIMUM study. Ultrasound Obstet Gynecol 48: 308–317. doi: 10.1002/uog.15953 27444208 10.1002/uog.15953 PMC5053235

[5] Romero R, Yeo L, Chaemsaithong P, Chaiworapongsa T, Hassan SS (2014) Progesterone to prevent spontaneous preterm birth. Semin Fetal Neonatal Med 19: 15–26. doi: 10.1016/j.siny.2013.10.004 24315687 10.1016/j.siny.2013.10.004 PMC3934502

[6] Romero R, Yeo L, Miranda J, Hassan SS, Conde-Agudelo A, et al (2013) A blueprint for the prevention of preterm birth: vaginal progesterone in women with a short cervix. J Perinat Med 41: 27–44. doi: 10.1515/jpm-2012-0272 23314512 10.1515/jpm-2012-0272 PMC4151573

[7] Alfirevic Z, Owen J, Carreras Moratonas E, Sharp AN, Szychowski JM, et al (2013) Vaginal progesterone, cerclage or cervical pessary for preventing preterm birth in asymptomatic singleton pregnant women with a history of preterm birth and a sonographic short cervix. Ultrasound Obstet Gynecol 41: 146–151. doi: 10.1002/uog.12300 22991337 10.1002/uog.12300

[8] Berghella V, Rafael TJ, Szychowski JM, Rust OA, Owen J (2011) Cerclage for short cervix on ultrasonography in women with singleton gestations and previous preterm birth: a meta-analysis. Obstet Gynecol 117: 663–671. 21446209 10.1097/AOG.0b013e31820ca847

[9] Owen J, Hankins G, Iams JD, Berghella V, Sheffield JS, et al (2009) Multicenter randomized trial of cerclage for preterm birth prevention in high-risk women with shortened midtrimester cervical length. Am J Obstet Gynecol 201: 375 e371–378. 19788970 10.1016/j.ajog.2009.08.015 PMC2768604

[10] Owen J, Mancuso M (2012) Cervical cerclage for the prevention of preterm birth. Obstet Gynecol Clin North Am 39: 25–33. doi: 10.1016/j.ogc.2011.12.001 22370105 10.1016/j.ogc.2011.12.001

[11] Szychowski JM, Berghella V, Owen J, Hankins G, Iams JD, et al (2012) Cerclage for the prevention of preterm birth in high risk women receiving intramuscular 17-alpha-hydroxyprogesterone caproate. J Matern Fetal Neonatal Med 25: 2686–2689. doi: 10.3109/14767058.2012.717128 22889234 10.3109/14767058.2012.717128 PMC3991237

[12] Wood SL, Owen J (2016) Cerclage: Shirodkar, McDonald, and Modifications. Clin Obstet Gynecol 59: 302–310. doi: 10.1097/GRF.0000000000000190 26974218 10.1097/GRF.0000000000000190

[13] Wood SL, Owen J (2016) Vaginal Cerclage: Preoperative, Intraoperative, and Postoperative Management. Clin Obstet Gynecol 59: 270–285. doi: 10.1097/GRF.0000000000000191 26974217 10.1097/GRF.0000000000000191

[14] Barinov SV, Shamina IV, Lazareva OV, Tirskaya YI, Ralko VV, et al (2016) Comparative assessment of arabin pessary, cervical cerclage and medical management for preterm birth prevention in high-risk pregnancies. J Matern Fetal Neonatal Med: 1–6. 10.1080/14767058.2016.1228054 27550418

[15] Goya M, de la Calle M, Pratcorona L, Merced C, Rodo C, et al (2016) Cervical pessary to prevent preterm birth in women with twin gestation and sonographic short cervix: a multicenter randomized controlled trial (PECEP-Twins). Am J Obstet Gynecol 214: 145–152. doi: 10.1016/j.ajog.2015.11.012 26627728 10.1016/j.ajog.2015.11.012

[16] Markham KB, Klebanoff M (2014) Prevention of preterm birth in modern obstetrics. Clin Perinatol 41: 773–785. doi: 10.1016/j.clp.2014.08.003 25459773 10.1016/j.clp.2014.08.003

[17] Sentilhes L, Senat MV, Ancel PY, Azria E, Benoist G, et al (2016) Prevention of spontaneous preterm birth: Guidelines for clinical practice from the French College of Gynaecologists and Obstetricians (CNGOF). Eur J Obstet Gynecol Reprod Biol 210: 217–224. doi: 10.1016/j.ejogrb.2016.12.035 28068594 10.1016/j.ejogrb.2016.12.035

[18] Martin JA, Hamilton BE, Osterman MJ, Curtin SC, Matthews TJ (2015) Births: final data for 2013. Natl Vital Stat Rep 64: 1–65. 25603115

[19] Henderson JJ, McWilliam OA, Newnham JP, Pennell CE (2012) Preterm birth aetiology 2004–2008. Maternal factors associated with three phenotypes: spontaneous preterm labour, preterm pre-labour rupture of membranes and medically indicated preterm birth. J Matern Fetal Neonatal Med 25: 642–647. doi: 10.3109/14767058.2011.597899 21827362 10.3109/14767058.2011.597899

[20] Romero R, Dey SK, Fisher SJ (2014) Preterm labor: one syndrome, many causes. Science 345: 760–765. doi: 10.1126/science.1251816 25124429 10.1126/science.1251816 PMC4191866

[21] Gotsch F, Gotsch F, Romero R, Erez O, Vaisbuch E, et al (2009) The preterm parturition syndrome and its implications for understanding the biology, risk assessment, diagnosis, treatment and prevention of preterm birth. J Matern Fetal Neonatal Med 22 Suppl 2: 5–23. 19951079 10.1080/14767050902860690

[22] Romero R, Espinoza J, Kusanovic JP, Gotsch F, Hassan S, et al (2006) The preterm parturition syndrome. BJOG 113 Suppl 3: 17–42. 10.1111/j.1471-0528.2006.01120.x PMC7062298 17206962

[23] Harteman JC, Nikkels PG, Kwee A, Groenendaal F, de Vries LS (2012) Patterns of placental pathology in preterm infants with a periventricular haemorrhagic infarction: association with time of onset and clinical presentation. Placenta 33: 839–844. doi: 10.1016/j.placenta.2012.06.014 22835681 10.1016/j.placenta.2012.06.014

[24] Liu Z, Tang Z, Li J, Yang Y (2014) Effects of placental inflammation on neonatal outcome in preterm infants. Pediatr Neonatol 55: 35–40. doi: 10.1016/j.pedneo.2013.05.007 23899553 10.1016/j.pedneo.2013.05.007

[25] Redline RW (2007) Infections and other inflammatory conditions. Semin Diagn Pathol 24: 5–13. 17455857 10.1053/j.semdp.2007.01.001

[26] Redline RW (2015) Classification of placental lesions. Am J Obstet Gynecol 213: S21–28. doi: 10.1016/j.ajog.2015.05.056 26428500 10.1016/j.ajog.2015.05.056

[27] Redline RW (2015) The clinical implications of placental diagnoses. Semin Perinatol 39: 2–8. doi: 10.1053/j.semperi.2014.10.002 25455619 10.1053/j.semperi.2014.10.002

[28] Soraisham AS, Trevenen C, Wood S, Singhal N, Sauve R (2013) Histological chorioamnionitis and neurodevelopmental outcome in preterm infants. J Perinatol 33: 70–75. doi: 10.1038/jp.2012.49 22555781 10.1038/jp.2012.49

[29] Kim CJ, Romero R, Chaemsaithong P, Chaiyasit N, Yoon BH, et al (2015) Acute chorioamnionitis and funisitis: definition, pathologic features, and clinical significance. Am J Obstet Gynecol 213: S29–52. doi: 10.1016/j.ajog.2015.08.040 26428501 10.1016/j.ajog.2015.08.040 PMC4774647

[30] Kim CJ, Romero R, Chaemsaithong P, Kim JS (2015) Chronic inflammation of the placenta: definition, classification, pathogenesis, and clinical significance. Am J Obstet Gynecol 213: S53–69. doi: 10.1016/j.ajog.2015.08.041 26428503 10.1016/j.ajog.2015.08.041 PMC4782598

[31] Kim CJ, Romero R, Kusanovic JP, Yoo W, Dong Z, et al (2010) The frequency, clinical significance, and pathological features of chronic chorioamnionitis: a lesion associated with spontaneous preterm birth. Mod Pathol 23: 1000–1011. doi: 10.1038/modpathol.2010.73 20348884 10.1038/modpathol.2010.73 PMC3096929

[32] Romero R, Gomez R, Ghezzi F, Yoon BH, Mazor M, et al (1998) A fetal systemic inflammatory response is followed by the spontaneous onset of preterm parturition. Am J Obstet Gynecol 179: 186–193. 9704786 10.1016/s0002-9378(98)70271-6

[33] Gomez R, Romero R, Ghezzi F, Yoon BH, Mazor M, et al (1998) The fetal inflammatory response syndrome. Am J Obstet Gynecol 179: 194–202. 9704787 10.1016/s0002-9378(98)70272-8

[34] Gotsch F, Romero R, Kusanovic JP, Mazaki-Tovi S, Pineles BL, et al (2007) The fetal inflammatory response syndrome. Clin Obstet Gynecol 50: 652–683. doi: 10.1097/GRF.0b013e31811ebef6 17762416 10.1097/GRF.0b013e31811ebef6

[35] Yoon BH, Romero R, Park JS, Kim M, Oh SY, et al (2000) The relationship among inflammatory lesions of the umbilical cord (funisitis), umbilical cord plasma interleukin 6 concentration, amniotic fluid infection, and neonatal sepsis. Am J Obstet Gynecol 183: 1124–1129. doi: 10.1067/mob.2000.109035 11084553 10.1067/mob.2000.109035

[36] Erdemir G, Kultursay N, Calkavur S, Zekioglu O, Koroglu OA, et al (2013) Histological chorioamnionitis: effects on premature delivery and neonatal prognosis. Pediatr Neonatol 54: 267–274. doi: 10.1016/j.pedneo.2013.03.012 23639744 10.1016/j.pedneo.2013.03.012

[37] Hirvonen M, Ojala R, Korhonen P, Haataja P, Eriksson K, et al (2014) Cerebral palsy among children born moderately and late preterm. Pediatrics 134: e1584–1593. doi: 10.1542/peds.2014-0945 25422011 10.1542/peds.2014-0945

[38] Mathiasen R, Hansen BM, Andersen AM, Forman JL, Greisen G (2010) Gestational age and basic school achievements: a national follow-up study in Denmark. Pediatrics 126: e1553–1561. doi: 10.1542/peds.2009-0829 21059721 10.1542/peds.2009-0829

[39] Saigal S, Doyle LW (2008) An overview of mortality and sequelae of preterm birth from infancy to adulthood. Lancet 371: 261–269. doi: 10.1016/S0140-6736(08)60136-1 18207020 10.1016/S0140-6736(08)60136-1

[40] Yoon BH, Romero R, Park JS, Kim CJ, Kim SH, et al (2000) Fetal exposure to an intra-amniotic inflammation and the development of cerebral palsy at the age of three years. Am J Obstet Gynecol 182: 675–681. 10739529 10.1067/mob.2000.104207

[41] Olischar M, Klebermass K, Kuhle S, Hulek M, Kohlhauser C, et al (2004) Reference values for amplitude-integrated electroencephalographic activity in preterm infants younger than 30 weeks' gestational age. Pediatrics 113: e61–66. 14702497 10.1542/peds.113.1.e61

[42] Klebermass K, Kuhle S, Olischar M, Rucklinger E, Pollak A, et al (2006) Intra- and extrauterine maturation of amplitude-integrated electroencephalographic activity in preterm infants younger than 30 weeks of gestation. Biol Neonate 89: 120–125. doi: 10.1159/000088912 16219998 10.1159/000088912

[43] Kuint J, Turgeman A, Torjman A, Maayan-Metzger A (2007) Characteristics of amplitude-integrated electroencephalogram in premature infants. J Child Neurol 22: 277–281. doi: 10.1177/0883073807299860 17621496 10.1177/0883073807299860

[44] Lamblin MD, Andre M, Challamel MJ, Curzi-Dascalova L, d'Allest AM, et al (1999) [Electroencephalography of the premature and term newborn. Maturational aspects and glossary]. Neurophysiol Clin 29: 123–219. 10367287 10.1016/s0987-7053(99)80051-3

[45] Klebermass K, Olischar M, Waldhoer T, Fuiko R, Pollak A, et al (2011) Amplitude-integrated EEG pattern predicts further outcome in preterm infants. Pediatr Res 70: 102–108. doi: 10.1203/PDR.0b013e31821ba200 21436758 10.1203/PDR.0b013e31821ba200

[46] Song J, Xu F, Wang L, Gao L, Guo J, et al (2015) Early amplitude-integrated electroencephalography predicts brain injury and neurological outcome in very preterm infants. Sci Rep 5: 13810 doi: 10.1038/srep13810 26348553 10.1038/srep13810 PMC4562298

[47] Wikstrom S, Pupp IH, Rosen I, Norman E, Fellman V, et al (2012) Early single-channel aEEG/EEG predicts outcome in very preterm infants. Acta Paediatr 101: 719–726. doi: 10.1111/j.1651-2227.2012.02677.x 22530996 10.1111/j.1651-2227.2012.02677.x PMC3437495

[48] Phelan JP, Smith CV, Broussard P, Small M (1987) Amniotic fluid volume assessment with the four-quadrant technique at 36–42 weeks' gestation. J Reprod Med 32: 540–542. 3305930

[49] Pri-Paz S, Khalek N, Fuchs KM, Simpson LL (2012) Maximal amniotic fluid index as a prognostic factor in pregnancies complicated by polyhydramnios. Ultrasound Obstet Gynecol 39: 648–653. doi: 10.1002/uog.10093 21898637 10.1002/uog.10093

[50] Mercer BM (2004) Preterm premature rupture of the membranes: diagnosis and management. Clin Perinatol 31: 765–782, vi. doi: 10.1016/j.clp.2004.06.004 15519427 10.1016/j.clp.2004.06.004

[51] Gibbs RS, Castillo MS, Rodgers PJ (1980) Management of acute chorioamnionitis. Am J Obstet Gynecol 136: 709–713. 7355955 10.1016/0002-9378(80)90445-7

[52] Mercer BM, Miodovnik M, Thurnau GR, Goldenberg RL, Das AF, et al (1997) Antibiotic therapy for reduction of infant morbidity after preterm premature rupture of the membranes. A randomized controlled trial. National Institute of Child Health and Human Development Maternal-Fetal Medicine Units Network. JAMA 278: 989–995. 9307346

[53] Redline RW, Heller D, Keating S, Kingdom J (2005) Placental diagnostic criteria and clinical correlation—a workshop report. Placenta 26 Suppl A: S114–117. 15837060 10.1016/j.placenta.2005.02.009

[54] Fenton TR, Kim JH (2013) A systematic review and meta-analysis to revise the Fenton growth chart for preterm infants. BMC Pediatr 13: 59 doi: 10.1186/1471-2431-13-59 23601190 10.1186/1471-2431-13-59 PMC3637477

[55] Amiel-Tison C (1968) Neurological evaluation of the maturity of newborn infants. Arch Dis Child 43: 89–93. 5689329 10.1136/adc.43.227.89 PMC2019902

[56] Amiel-Tison C (1976) A method for neurologic evaluation within the first year of life. Curr Probl Pediatr 7: 1–50. 975863

[57] Amiel-Tison C (1978) A method for neurological evaluation within the first year of life: experience with full-term newborn infants with birth injury. Ciba Found Symp: 107–137. 10.1002/9780470720417.ch7 251500

[58] Amiel-Tison C (2002) Update of the Amiel-Tison neurologic assessment for the term neonate or at 40 weeks corrected age. Pediatr Neurol 27: 196–212. 12393130 10.1016/s0887-8994(02)00436-8

[59] Aylward GP (2004) Prediction of function from infancy to early childhood: implications for pediatric psychology. J Pediatr Psychol 29: 555–564. doi: 10.1093/jpepsy/jsh057 15347703 10.1093/jpepsy/jsh057

[60] Aylward GP, Verhulst SJ (2000) Predictive utility of the Bayley Infant Neurodevelopmental Screener (BINS) risk status classifications: clinical interpretation and application. Dev Med Child Neurol 42: 25–31. 10665972 10.1017/s0012162200000062

[61] Report of the committee on methods of clinical examination in electroencephalography. Electroencephalography and Clinical Neurophysiology 10: 370–375.

[62] Maynard D, Prior PF, Scott DF (1969) A continuous monitoring device for cerebral activity. Electroencephalogr Clin Neurophysiol 27: 672–673. 10.1016/0013-4694(69)91265-6 4187315

[63] Prior PF, Maynard DE, Sheaff PC, Simpson BR, Strunin L, et al (1971) Monitoring cerebral function: clinical experience with new device for continuous recording of electrical activity of brain. Br Med J 2: 736–738. 4326286 10.1136/bmj.2.5764.736 PMC1796304

[64] Hellström-Westas L, Rosén I, de Vries LS, Greisen G (2006) Amplitude-integrated EEG Classification and Interpretation in Preterm and Term Infants. NeoReviews 7: e76–e87.

[65] Lee J, Romero R, Chaiworapongsa T, Dong Z, Tarca AL, et al (2013) Characterization of the fetal blood transcriptome and proteome in maternal anti-fetal rejection: evidence of a distinct and novel type of human fetal systemic inflammatory response. Am J Reprod Immunol 70: 265–284. doi: 10.1111/aji.12142 23905683 10.1111/aji.12142 PMC3939790

[66] Romero R, Miranda J, Chaiworapongsa T, Korzeniewski SJ, Chaemsaithong P, et al (2014) Prevalence and clinical significance of sterile intra-amniotic inflammation in patients with preterm labor and intact membranes. Am J Reprod Immunol 72: 458–474. doi: 10.1111/aji.12296 25078709 10.1111/aji.12296 PMC4192099

[67] Adams-Chapman I, Vaucher YE, Bejar RF, Benirschke K, Baergen RN, et al (2002) Maternal floor infarction of the placenta: association with central nervous system injury and adverse neurodevelopmental outcome. J Perinatol 22: 236–241. doi: 10.1038/sj.jp.7210685 11948388 10.1038/sj.jp.7210685

[68] Moscuzza F, Belcari F, Nardini V, Bartoli A, Domenici C, et al (2011) Correlation between placental histopathology and fetal/neonatal outcome: chorioamnionitis and funisitis are associated to intraventricular haemorrage and retinopathy of prematurity in preterm newborns. Gynecol Endocrinol 27: 319–323. doi: 10.3109/09513590.2010.487619 20528214 10.3109/09513590.2010.487619

[69] Redline RW (2005) Severe fetal placental vascular lesions in term infants with neurologic impairment. Am J Obstet Gynecol 192: 452–457. doi: 10.1016/j.ajog.2004.07.030 15695986 10.1016/j.ajog.2004.07.030

[70] Redline RW, Minich N, Taylor HG, Hack M (2007) Placental lesions as predictors of cerebral palsy and abnormal neurocognitive function at school age in extremely low birth weight infants (<1 kg). Pediatr Dev Pathol 10: 282–292 17638433 10.2350/06-12-0203.1

[71] Roescher AM, Timmer A, Erwich JJ, Bos AF (2014) Placental pathology, perinatal death, neonatal outcome, and neurological development: a systematic review. PLoS One 9: e89419 doi: 10.1371/journal.pone.0089419 24586764 10.1371/journal.pone.0089419 PMC3934891

[72] Lee J, Kim JS, Park JW, Park CW, Park JS, et al (2013) Chronic chorioamnionitis is the most common placental lesion in late preterm birth. Placenta 34: 681–689. doi: 10.1016/j.placenta.2013.04.014 23684379 10.1016/j.placenta.2013.04.014

[73] Nijman TA, van Vliet EO, Benders MJ, Mol BW, Franx A, et al (2016) Placental histology in spontaneous and indicated preterm birth: A case control study. Placenta 48: 56–62. doi: 10.1016/j.placenta.2016.10.006 27871473 10.1016/j.placenta.2016.10.006

